# *WordCluster*: detecting clusters of DNA words and genomic elements

**DOI:** 10.1186/1748-7188-6-2

**Published:** 2011-01-24

**Authors:** Michael Hackenberg, Pedro Carpena, Pedro Bernaola-Galván, Guillermo Barturen, Ángel M Alganza, José L Oliver

**Affiliations:** 1 Dpto. de Genética, Facultad de Ciencias, Universidad de Granada, Campus de Fuentenueva s/n, 18071-Granada & Lab. de Bioinformática, Centro de Investigación Biomédica, PTS, Avda. del Conocimiento s/n, 18100-Granada, Spain; 2 Dpto. de Física Aplicada II, E.T.S.I. de Telecomunicación, Universidad de Málaga 29071-Malaga, Spain; 3 Division of Sleep Medicine, Brigham and Woman's Hospital, Harvard Medical School, Boston, MA 02115, USA

## Abstract

**Background:**

Many *k-*mers (or DNA words) and genomic elements are known to be spatially clustered in the genome. Well established examples are the genes, TFBSs, CpG dinucleotides, microRNA genes and ultra-conserved non-coding regions. Currently, no algorithm exists to find these clusters in a statistically comprehensible way. The detection of clustering often relies on densities and sliding-window approaches or arbitrarily chosen distance thresholds.

**Results:**

We introduce here an algorithm to detect clusters of DNA words (*k-*mers), or any other genomic element, based on the distance between consecutive copies and an assigned statistical significance. We implemented the method into a web server connected to a MySQL backend, which also determines the co-localization with gene annotations. We demonstrate the usefulness of this approach by detecting the clusters of CAG/CTG (cytosine contexts that can be methylated in undifferentiated cells), showing that the degree of methylation vary drastically between inside and outside of the clusters. As another example, we used *WordCluster *to search for statistically significant clusters of olfactory receptor (OR) genes in the human genome.

**Conclusions:**

*WordCluster *seems to predict biological meaningful clusters of DNA words (*k-*mers) and genomic entities. The implementation of the method into a web server is available at http://bioinfo2.ugr.es/wordCluster/wordCluster.php including additional features like the detection of co-localization with gene regions or the annotation enrichment tool for functional analysis of overlapped genes.

## Background

Genome entities as diverse as genes [1], CpG dinucleotides [2], transcription factor binding sites (TFBSs [3]) or ultra-conserved non-coding regions [4] usually form clusters along the chromosome sequence. Such spatial clustering often translates into genome structures with a clear functional and/or evolutionary meaning: gene clusters encoding the same or similar products and originated through gene duplication events, CpG islands, cis-regulatory modules, etc. Thus, the spatial clustering of functional genome elements (in general, words or *k-*mers) would somewhat remember the situation in literary texts, where keywords show a strong clustering, whereas common words are randomly distributed [5].

Despite its potential importance, no algorithm exists to detect the clustering of DNA words in a rigorous way. Most current methods are based on densities and sliding-window approaches or arbitrary distances. For example, the Galaxy work suite ([6], http://main.g2.bx.psu.edu/) implements an algorithm which lets the user decide to fix the maximum distance between two entities and the minimum number of entities in the cluster. Recently, we developed an algorithm to detect clusters of CpG dinucleotides in DNA sequences based on the distance between neighboring CpGs, then assigning a statistical significance [7]. Now, we generalize the method to any *k-*mer or any arbitrary combination of them, as well as to any other genome entity defined by its chromosome coordinates.

## Implementation

The *WordCluster *algorithm allows the detection of clusters for DNA words (*k-*mers) and genomic elements (genes, transposons, SINEs, TFBSs, etc.). The algorithm is based on the distances between the entities and an assigned *p-value*.

### The algorithm

The algorithm is basically the same for *k-*mers and genomic elements except for the detection of the coordinates and the way the success probabilities are calculated. Briefly the algorithm performs the following steps:

1. Detection of all *k-*mer copies in the chromosomes, storing its coordinates (this step is unique to the detection of *k-*mer clusters as the genomic elements already come defined by its coordinates). The copies are detected in a non-overlapping way, i.e. once a copy is found the search is resumed at the end of the word, thus preventing the detection of overlapping copies.

2. Calculation of the distances between consecutive copies. The distance is defined as: "start coordinate of the downstream copy" minus "end coordinate of the upstream copy". This implies that the minimum distance is 1 when the two entities are located directly next to each other.

3. Detection of the clusters, defined as those chromosomal regions where all distances are equal or below a given maximum distance. A cluster is defined by its start and end coordinates and the number of *k-*mers or genomic elements it contain.

4. Calculation of the statistical significance for each cluster by means of the negative binomial distribution. A *p-value *threshold is then used to filter out those clusters which are not statistically significant.

A main difference to the originally described algorithm is the way N-runs in the DNA sequence (ambiguous sequence sites occupied by any nucleotide) are treated. While the original *CpGcluster *method allows up to 10 Ns between two consecutive CpGs, *WordCluster *detects the DNA words and the distances strictly within the contigs, i.e. not a single N is allowed to lie between two copies.

### Statistical significance

From now on, we will have to use the word *k*-mer in different contexts. Therefore, to avoid confusion we define as "target *k*-mer(s)" the *k*-mer(s) which are being analysed, i.e. those for which the clusters are going to be detected. On the contrary, "no-target *k*-mer(s)" are all the remaining *k*-mer(s). We use *k*-mer in a generic way, referring to all DNA words of length *k*.

The statistical significance is calculated as the cumulative density function of the negative binomial distribution:

$$P_{N,p}(n_{f})=\left(\begin{array}{c} n_{f}+(n-1)-1\\ (n-1)-1 \end{array}\right)\cdot p^{n-1}\cdot {\left(1-p\right)}^{n_{f}}$$

being *n *the number of target *k-*mers within the cluster, *n*_*f *_the number of "failures", i.e. the number of no-target *k*-mers. For example, if we are detecting clusters of AGCT, all *k-*mers other than AGCT would be considered as failures. Finally, *p *is the success probability, i.e. the probability to find a target *k-*mer or genomic element within the DNA sequence. Note that in the above equation we use (*n*-1) instead of *n*, as the first appearance of a target *k*-mer within the cluster is trivial (i.e. all the clusters start with a target *k*-mer). While the negative binomial distribution can be defined in the same way for *k-*mers and genomic elements, differences exist in the way the number of "failures" and the success probability are calculated.

For *k-*mers, the number of failures *n*_*f *_is simply given by

$$n_{f}=L_{c}-n\cdot k$$

being *L*_*c *_the length of the cluster, *k *the length of the target *k-*mer and *n *the number of non-overlapping target *k*-mers in the cluster. The number of failures is the number of no-target *k*-mers within the cluster. For example, given the target *k*-mer ATGC, the cluster ATGCATGC would give *n*_*f *_= 0 while ATGCAATGC would give *n*_*f *_= 1. Each *k-*mer can overlap with itself and other *k-*mers, but here we consider just non-overlapping occurrences. In such a case, the probabilities for *k-*mers are given by the following equation

$$p=\frac{N}{(L_{s}-k+1)-N\cdot \left(k-1\right)}$$

being *N *the number of non-overlapping occurrences of the target *k-*mers in the sequence, *k *the length of the *k-*mer and *L*_*s *_the sequence length. The formula is simply the number of target *k*-mers in the sequence divided by the total number of *k*-mers in the sequence. As we do not consider overlapping instances, N*(*k*-1) was subtracted from the total number of *k*-mers (*L*_*s *_- *k *+ 1), as those sequence positions are not considered, in order to take this effect into account.

For genomic elements, it is less clear how to define the number of failures. For example, one has a cluster with 5 elements which have mean length of 300 bp and 250 bp of distance on average between each other. The question is how many "no-elements" contain this cluster, i.e. how many failures. We define the number of failures as

$$n_{f}=ceiling(\frac{L_{no}}{L_{mean}})$$

being *L*_*no *_the number of bases in the cluster not belonging to the genomic element and *L*_*mean *_the mean length of the genomic element. Thus, this number is an approximation to the number of "no-elements" within the cluster. Finally, the success probability is then given as

$$p=\frac{N\cdot L_{mean}}{L_{S}}$$

being *L*_*s *_the length of the sequence, *L*_*mean *_the mean length of the genomic elements and *N *the number of genomic elements.

### Distance models

**Figure 1:**
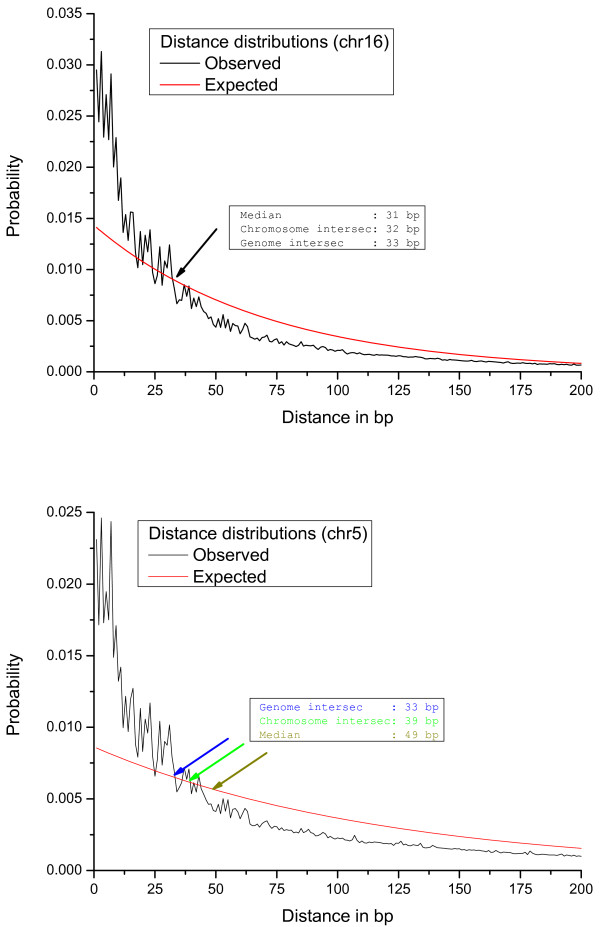
**Distance distributions**. Expected and observed distance distributions for human chromosomes 16 (above) and 5 (below). It can be seen that for chr16 the median, the chromosome intersection and the genome intersection are very close (within 1 bp), while for chromosome 5 notable differences exist (from 33 bp to 49 bp).

The maximum distance is the main parameter of the algorithm determining the copies belonging to each cluster. We have shown previously [7] that, for most human chromosomes, the median of the observed distance distribution of CpGs lies near the intersection between the observed and the expected distance distribution. The intersection can be interpreted as the point separating the intra-cluster from the inter-cluster distances. In this new tool, we added two more distance models based on the direct detection of the mentioned intersection (one genome wide and the other for each chromosome separately). In this way, *WordCluster *implements a total of 4 different distance models:

1. Percentile distance: The distance corresponding to a given percentile of the observed distance distribution is calculated and used as the maximum distance threshold.

2. Chromosomal intersection: The distance corresponding to the intersection between the observed and the expected distributions is used as the maximum distance (see Figure 1).

3. Genome intersection: The distance distributions for all chromosomes are merged, then calculating the distance corresponding to the "genome intersection point". If this distance model is chosen, the success probabilities (i.e. the probability to find the target *k-*mers in the chromosome) are not calculated for each chromosome separately (like in the two models above), but a genome wide success probability (probability to find the target *k*-mers) is calculated.

4. Fixed distance: the user can set the distance threshold.

### Webserver

We implemented the described algorithm into a web server. The tool uses PHP for the interaction with the user, to access the core program (written in Java) and the MySQL database. Two types of input data can be supplied: 1) a group of *k-*mers and a genomic sequence to be scanned by the program (the user can upload his own sequence or choose one of the 24 genome assemblies stored in our database - see below); and 2) a file in BED format [8,9] with the coordinates of the genomic elements whose clustering properties should be analyzed. No mandatory input parameters exist, but the user can select between different distance models (the default is the chromosome intersection) and set the cut-off for the statistical significance (the default here is *p-value *≤ 1E-5).

The output generated by the web server depends on whether the user chooses a genome assembly from our database or supplies an anonymous sequence. The minimum output consists of the basic statistics of the clusters (base composition, entity composition and statistical significance) and the statistics by chromosome. Furthermore, for all species in the database, the co-localization of detected clusters with different gene regions (promoters, introns, etc.) is reported.

Finally, for some species (human, mouse, rat, cow, *C. elegans*, zebrafish and chicken) an enrichment/depletion analysis for the genes overlapped by the clusters is carried out using the Gene Ontology [10] and the Annotation-Modules database [11,12].

### Database

Currently, the genomes of 24 genome assemblies are stored into our database. The following sequences where downloaded from the UCSC genome browser or the corresponding project homepages (plant genomes): Human (hg18, hg19), Mouse (mm8, mm9), Rat (rn4), Fruit fly (dm3), *Anopheles gambiae *(anogam1), Honey bee (apimel2), Cow (bosTau4), Dog (canFam2), *C. briggsae *(cb3), *C. elegans *(ce6), Sea squirt (ci2), Zebrafish (danrer5), Chicken (galgal3), Stickleback (gasacu1), Medaka (orylat2), Chimp (pantro2), *Rhesus *macaque (rhemac2), *S. cerevisiae *(saccer1), *Tetraodon *(tetnig1), *Arabidopsis thaliana *(tair8, tair9), and *Zea mays *(zm1). To determine the co-localization with genes, we used RefSeq genes whenever they were available [13], Ensembl genes otherwise [14].

## Results and Discussion

To demonstrate the ability of our algorithm in finding biologically significant and relevant clusters in the genome, at the same time illustrating the different distance models, we carried out three analysis: 1) detection of clusters of CpGs (CpG islands) using different distance models, 2) detection of clusters of the word CWG (where W = A, T) and 3) detection of clusters of olfactory receptor genes in the human chromosome 11.

### Detection of CpG islands with different distance models

We choose this example as the detection of CpG islands was the reason to develop the algorithm from which *WordCluster *[7] was derived. In the original *CpGcluster *algorithm, we used the percentile of the observed distance distribution as distance model (apart from the fixed distance), suggesting the median as the default parameter. We did this since we observed that the intersection between the observed and expected distance distributions is often very close to the median of the observed distance distribution (see Figure 1). This intersection can be interpreted in the following way. When the observed curve lies above the expected, theoretical curve, it means that more CpGs exist at this distance than expected by chance. We can observe in Figure 1 that this is generally the case for short distances, thus indicating the clustering (overrepresentation of short distances) of CpG dinucleotides. The intersection defines the "reversal point", i.e. at larger distances than this point, the CpG dinucleotides are not clustered any more. Therefore, it might be that the strict use of the intersection defines better clusters that the use of the median, which is a mere approximation to the intersection point. Furthermore, we observed that for some chromosomes the intersection and the median differ slightly. To clarify the impact of this change in the maximum distance, we predict CpG islands by means of the median (cpg50), the chromosome intersection (cpgISc) and the genome intersection (cpgISg), then assessing the prediction quality by some of the criteria previously described [7,15]. Table 1 shows that the mean length of both intersection models are clearly below the mean length of the original cpg50 islands. This can be explained as the intersection models produce on average shorter distance thresholds, which leads to fragmentation, shortening and disappearance of some cpg50 CpG islands. Consequently, the chromosome intersection model (cpgISc) predicts fewer islands than the original cpg50 algorithm (3979). Nevertheless, the genome intersection (cpgISg) yields more predictions compared to cpg50 (5535). The latter observation can be explained as the predictions are done with a single, genome wide probability. The *p-value *assigned to each cluster depends on the success probability, and in G+C rich chromosomes the genome wide probability is much lower than the chromosome probability. This leads to smaller *p-value*s in G+C rich chromosomes, so that more islands can pass the *p-value *threshold. For example, cpg50 predicts 2434 islands in chromosome 22 while cpgISg predicts 5197. Of course, in AT-rich chromosomes this effect is reverted but less pronounced (the difference between genome wide and chromosome probabilities are smaller in AT-rich compared to GC-rich chromosomes), and therefore a higher total number of islands are predicted.

**Table 1:** *WordCluster *predictions of CpG clusters*

Method	#	Length ± SD	GC ± SD	OE ± SD
cpg50	198703	273.2 ± 246.4	63.8 ± 7.5	0.855 ± 0.265
cpgISc	194725	218.7 ± 200.1	65.6 ± 7.7	0.916 ± 0.273
cpgISg	204238	202.6 ± 183.8	66.3 ± 7.5	0.930 ± 0.274

*Basic statistic of CpG island predictions using three different distance models: cpgISg (genome intersection), cpg50 (Median) and cpgISc (chromosome intersection). The number of predicted islands, the length, the G+C content and the observed to expected ratios are shown. Note that the original cpg50 algorithm predicts 198702 islands, i.e. one less than *WordCluster *with the median model. This is due to the changes introduced regarding the N-runs (see main text).

Next, we analyzed the predictions under functional aspects. Table 2 shows the overlap of the predictions with RefSeq genes [13], Alu elements and phylogenetically conserved PhastCons elements [16]. The cpgISg predictions show the highest overlap with the promoter region (R13), and conserved PhastCons elements, simultaneously showing the lowest overlap with spurious Alu elements. This might indicate that cpgISg predictions are slightly better than the other two, the original cpg50 and cpgISc. However, 1) the differences seem to be rather small and 2) a more detailed analysis would be needed to resolve this question.

**Table 2:** Biological meaning of *WordCluster *predictions*

Method	#islands	#TSS overlap	#R13 overlap	#Alu overlap	#PhastCons overlap
cpg50	198703	12432 (6.3%)	30660 (15.4%)	80323 (40.4%)	48787 (24.6%)
cpgISc	194724	11926 (6.1%)	34567 (17.8%)	70144 (36.0%)	48930 (25.1%)
cpgISg	204238	12156 (6.0%)	37616 (18.4%)	70456 (34.5%)	52335 (25.6%)

*Comparison of three *WordCluster *predictions of CG clusters (CpG islands) using three different distance models: cpgISg (genome intersection), cpg50 (median) and cpgISc (chromosome intersection). The overlap with two gene regions (TSS and R13), Alu elements and phylogenetically conserved PhastCons elements have been measured and both absolute numbers and percentages are given.

Independently of this open question, we can summarize: 1) the chromosome intersection seems to be a good replacement for the median and furthermore removes one input parameter from the method, as the intersection is a fixed statistical property of the chromosome; 2) the genome intersection may be used when the expected clusters are known to be not dependent on the chromosome. The CpG islands are probably not dependent on the chromosome, as the biological mechanisms forming and maintaining them are probably the same for all chromosomes. This may suggest the use of the genome intersection, which is confirmed by producing slightly better results than the other two tested distance models.

### Detection of CWG clusters

Besides the conventional CpG context, the CWG context has recently been shown to be a potential target for methylation [17]. *WordCluster *detects 84996 CAG/CTG clusters in the human genome (NCBI 36, hg18) significant at the 1E-5 level using the chromosome intersection (Table 1). We found a high number of statistically significant CWG clusters scattered along all human chromosomes, many of which are overlapping gene regions (Table 3). To check if the detected clusters might be biologically meaningful, we compared the percentage of methylated words (CAG and CTG) inside and outside of the clusters. We observed that 26.7% of all CAG/CTG trinucleotides are methylated inside the clusters while 45.3% of them are methylated when located outside a cluster. It seems therefore, as occurs in CpG islands, that CAG/CTG clusters remain unmethylated with a much higher probability than the bulk DNA.

**Table 3:** Clusters of CWG trinucleotides*

N	84996
Genome coverage (bp)	15700789
Average length (bp)	184.7
No. of clusters co-locating with gene regions:	
TSS	272
TSS ± 100 bp	686
5'UTR	4712
Introns	29326
Exons	1852
3'UTR	1658

*Statistically significant clusters of CAG and CTG trinucleotides detected by *WordCluster *in the human genome (hg18). We used the "genome intersection" distance model and a *p-value *threshold of 1E-05.

### Detection of olfactory gene clusters

**Figure 2:**
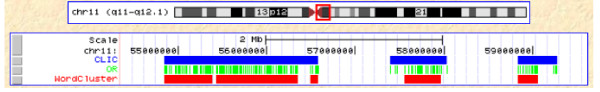
**Clusters of OR genes**. A region of human chromosome 11 showing OR genes (green), the clusters annotated in the CLIC/HORDE database (blue) and the statistically significant clusters predicted by *WordCluster *(red). Our algorithm predicts more compact clusters compared to the CLIC/HORDE annotation. For example, in the first and third HORDE clusters pronounced gaps exist between the genes, which is detected by *WordCluster *but ignored by the CLIC/HORDE annotation. The figure was generated using the UCSC Genome Browser [8].

As a third example, we used *WordCluster *to search for significant clusters of olfactory receptor (OR) genes, the largest multigene family in multicellular organisms whose members are known to be clustered within vertebrate genomes [18,19]. Table 4 shows the basic statistics for the 13 clusters of OR genes detected by our algorithm in human chromosome 11. Figure 2 shows a comparative analysis of the clusters predicted by *WordCluster *to the clusters currently annotated in the CLIC/HORDE database [19] in a selected region of chromosome 11. Our algorithm predicts a higher number of clusters, being all of them statistically significant.

**Table 4:** Clusters of OR genes in human chromosome 11*

Cluster	chromStart	chromEnd	length	count	*p-value*
1	4345160	5178488	833329	53	1.60E-49
2	5269273	5559687	290415	21	6.80E-21
3	5697096	6177989	480894	28	2.70E-25
4	48194938	48344593	149656	9	2.50E-08
5	48398372	48505102	106731	9	1.70E-09
6	49876392	49960613	84222	7	2.60E-07
7	51250039	51384376	134338	11	1.60E-11
8	54842612	55380573	537962	32	4.00E-29
9	55427396	56344568	917173	66	6.30E-65
10	56495101	56580184	85084	7	2.90E-07
11	57555001	57964200	409200	22	3.20E-19
12	58833691	59056759	223069	12	1.30E-10
13	123181329	123481891	300563	16	5.40E-14

*Chromosome coordinates, length, number of OR genes and *p-values *for all statistically significant OR gene clusters in chromosome 11.

## Conclusions

*WordCluster *generalizes the previous *CpGcluster *algorithm [7] to any word or genomic element in the genome, at the same time associating a statistical significance to the clusters found. It outperforms current methods relying on densities and sliding-window approaches or arbitrarily chosen distance thresholds. The implementation as a web server connected to a MySQL backend allows for co-localization studies with different gene regions, as well as for genome wide enrichment/depletion analysis of functional terms (GO).

## Availability and requirements

The *WordCluster *webserver (http://bioinfo2.ugr.es/wordCluster/wordCluster.php) is freely available. No registering is needed but every access is logged. For large jobs, a long-life web link to the results is provided.

## List of abbreviations used

*k-*mer: DNA word (oligonucleotide) with length *k; *SINEs: Short interspersed nuclear elements; TSS: Transcription Start Site; TFBS: Transcription Factor Binding Site; R13: promoter region [TSS-1500 bp; TSS+500 bp].

## Competing interests

None declared

## Authors' contributions

MH developed and implemented the algorithm and wrote the manuscript (with JLO), PC and PB carried out the theoretical analysis of word clustering and help with the interpretation of statistical results, GB and AMA retrieve and organize the genome and methylation databases, and JLO developed the algorithm and wrote the manuscript (with MH). All the authors critically read and approved the final version.
